# Bridging the organoid translational gap: integrating standardization and micropatterning for drug screening in clinical and pharmaceutical medicine

**DOI:** 10.1093/lifemedi/lnae016

**Published:** 2024-04-15

**Authors:** Haowei Yang, Jiawei Li, Zitian Wang, Davit Khutsishvili, Jiyuan Tang, Yu Zhu, Yongde Cai, Xiaoyong Dai, Shaohua Ma

**Affiliations:** Tsinghua Shenzhen International Graduate School (SIGS), Tsinghua University, Shenzhen 518055, China; Tsinghua-Berkeley Shenzhen Institute, Shenzhen 518055, China; Tsinghua Shenzhen International Graduate School (SIGS), Tsinghua University, Shenzhen 518055, China; Tsinghua-Berkeley Shenzhen Institute, Shenzhen 518055, China; Tsinghua Shenzhen International Graduate School (SIGS), Tsinghua University, Shenzhen 518055, China; Tsinghua Shenzhen International Graduate School (SIGS), Tsinghua University, Shenzhen 518055, China; Tsinghua Shenzhen International Graduate School (SIGS), Tsinghua University, Shenzhen 518055, China; Guangdong Research Center of Organoid Engineering and Technology, Guangzhou 510530, China; Tsinghua Shenzhen International Graduate School (SIGS), Tsinghua University, Shenzhen 518055, China; Tsinghua Shenzhen International Graduate School (SIGS), Tsinghua University, Shenzhen 518055, China; Tsinghua Shenzhen International Graduate School (SIGS), Tsinghua University, Shenzhen 518055, China; Tsinghua-Berkeley Shenzhen Institute, Shenzhen 518055, China; Key Laboratory of Industrial Biocatalysis (Ministry of Education), Tsinghua University, Beijing 100084, China

**Keywords:** organoid, organoid micropatterning, organoid standardization, organoids-on-a-chip

## Abstract

Synthetic organ models such as organoids and organ-on-a-chip have been receiving recognition from administrative agencies. Despite the proven success of organoids in predicting drug efficacy on laboratory scales, their translational advances have not fully satisfied the expectations for both clinical implementation and commercial applications. The transition from laboratory settings to clinical applications continues to encounter challenges. Employing engineering methodologies to facilitate the bridging of this gap for organoids represents one of the key directions for future advancement. The main measures to bridge the gap include environmental and phenotypic recapitulation, 3D patterning, matrix engineering, and multi-modality information acquisition and processing. Pilot whole-process clinical/pharmaceutical applications with fast and standardized organoid models will continuously offer convincing frontline optimization clues and driving forces to the organoid community, which is a promising path to translational organoid technologies.

## Introduction

Organoids are miniature *in vitro* models of organs that mimic the complexities of actual organs by providing stem cells with an array of biochemical and biophysical stimuli. These stimuli replicate the natural stem cell niche, enabling the organoids to maintain proliferation, differentiation abilities, and self-renewal capacities similar to their *in vivo* counterparts. In contrast to traditional 2D culture systems, 3D organoids more accurately reflect the attributes of native organs, including gene and protein expression profiles, metabolic functions, and histological structures [1]. Organoid technology is versatile and faithful, making it an invaluable and rapidly evolving tool with broad applications across clinical therapy and the pharmaceutical industry.

The evolution of modern medicine is progressively focusing on precision and personalization, particularly evident in the shift toward patient-specific treatments in precision medicine. This approach is crucial in cancer treatment, where the substantial heterogeneity of cancer cells undermines the one-size-fits-all therapies [2]. Precision medicine, therefore, aims to tailor treatments to the unique genetic, molecular, and cellular characteristics of an individual’s cancer, effectively addressing this variability. A key factor in this context is the tumor microenvironment, which significantly influences both cancer progression and prognostics. In this light, organoids become valuable tools for the study and development of targeted immunotherapies, as they closely mimic the cancer microenvironment. Similarly, in pharmaceutical development, traditional methods marked by high costs and lengthy timelines—screening thousands of compounds with few reaching clinical trials—are undergoing transformation [3]. In a historical context, as of 1996, most medicinal compounds were formulated to address approximately 500 molecular targets, with GTP-binding protein-coupled receptors constituting the largest group at 45%, followed by enzymes at 28% [4]. However, the current trend is shifting toward more specialized treatments, such as cellular and genetic therapies, antibodies, and biologics, which are attracting increasing focus [5]. These advanced therapeutic approaches necessitate more precise screening techniques, for which organoids, as accurate and versatile modeling systems, offer significant promise in the development of targeted pharmaceuticals [6, 7].

**Figure 1.**
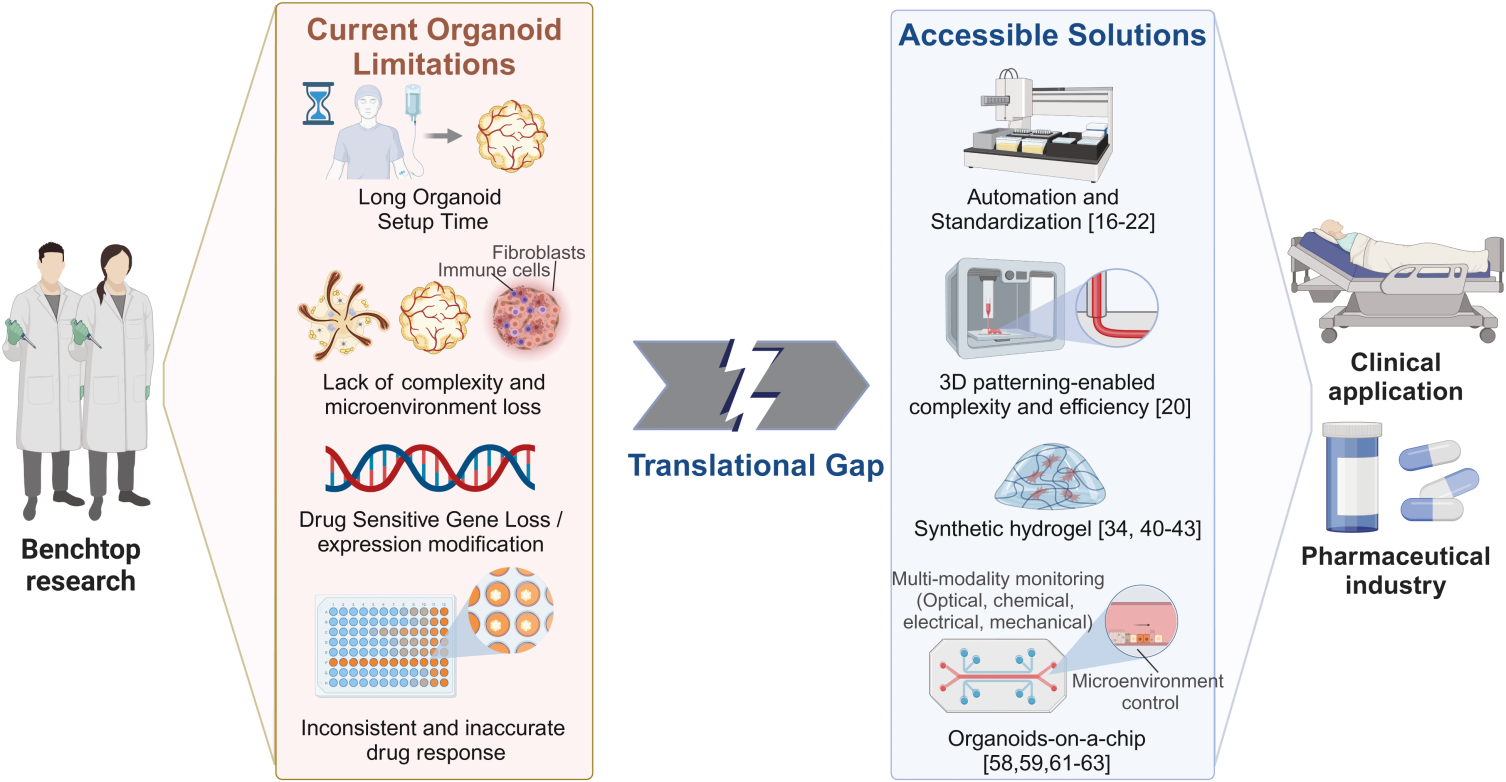
Current benchtop research organoid limitations and accessible solutions to bridging the gap.

Synthetic *in vitro* organ models have gained increasing recognition from regulatory agencies such as the US Food and Drug Administration (FDA) and China’s Center for Drug Evaluation since late 2021, organoids are officially recognized as an alternative to animals before human trails by the FDA. The organoid community has made significant progress in demonstrating the high accuracy of patient-derived organoids at the laboratory scale, particularly in predicting individual responses to clinical treatments. However, challenges remained in heterogeneity, reproducibility, and the need for clinical validation. Despite the substantial investment of nearly one billion US dollars in organoid technology, there remains a significant gap between laboratory research and practical clinical applications (Fig. 1). This disparity is evident in the limited direct benefits that organoid-based therapies and drugs have brought to patients. While the world sees approximately 20 million new cancer cases each year, organoid-based screening methods are limited to fewer than 20,000 patients. In the field of pharmaceuticals, there is a significant lack of drugs developed or validated through organoid technology. Pharmaceutical companies such as Roche, Novartis, and AstraZeneca have shown interest in organoid technology. However, the revenue of the organoid industry is less than 2 million dollars [8]. This highlights the potential for growth in integrating organoid-based services in clinical applications and drug development.

The persisting translational gap can be attributed to multiple factors. One primary challenge is the lack of standardized and rapid organoid fabrication, which would facilitate quick and extensive screening of numerous compounds for pharmaceuticals. These models are also crucial for the timely evaluation of patient drug efficacy. Additionally, there is an urgent need for novel organoid fabrication and characterization infrastructures. The integrated bioprinter can increase the complexity of organoids and greatly reduce variability. The combination of automated and dynamic culture techniques has led to the development of “organoids-on-a-chip” technology. This application enables automated dynamic culture and multi-modal data access on small microfluidic chips. Another significant challenge is matrix material. Current organoids still rely on expensive animal-derived extracellular matrix (ECM). However, one-fits-all matrices are apparently inadequate to meet the precise requirements of different patients and different types of organoids.

## Rapid modeling and standardization are critical to translational organoid technologies

A classical technique for generating epithelial and tumor organoids involves suspending stem or primary cells in a basement membrane matrix, followed by aliquoting a portion of this suspension into culture dishes or wells [9, 10]. However, this method often faces challenges in terms of control, reproducibility, and the absence of a stromal microenvironment, which are essential for producing consistent and physiologically relevant organoids [11]. Furthermore, the success of patient-derived organoid-based drug screening in personalized cancer care depends on a quick turnaround time from tumor sampling to drug recommendation. Classic organoids, derived from primary tumor tissues, traditionally require several weeks to develop sufficiently for drug screening, a timeline not always feasible for patient care [9]. To expedite this process, Xi et al. [12] introduced a 14-day drug evaluation protocol using tumor cell clusters, facilitating quicker organoid formation and drug testing. Subsequent research has focused on reducing the time required to develop organoids, particularly post-primary cell culture, with reports of successful organoid growth within one week of cell seeding. It has also been established that, in addition to appropriate niche factor guidance, non-chemical factors like mechano-stimulation, stromal microenvironment support, and matrix microstructure are crucial for the rapid maturation of organoids.

For effective translation in medical applications, organoid modeling must meet several criteria. Rapid development is crucial, ideally within one week, to facilitate personalized medicine screening. The process should utilize primary tissue cells directly, avoiding the need for extensive *in vitro* expansion of cancer stem cells. Standardization is key, ensuring reproducible organoid formation and personnel-independent procedures. Scalability is also essential, with the capacity to produce over 100,000 units in a few weeks for drug candidate evaluations and treatment combinations. Organoid customization in terms of size, shape, and cell composition is critical, as these factors influence cell metabolism, regulatory environments, and stem cell outcomes. Once established, production parameters should yield consistent results. Disease and treatment modeling, including for tumors and tissue fibrosis, requires co-culturing parenchyma cells with environmental cells such as fibroblasts, macrophages, and T cells. Proximity is crucial; a recent study indicates that on a 2D surface, a 100 μm distance between cancer cells and lymphocytes impacts their interaction [13]. This critical distance is likely reduced in 3D environments.

Recently, colonic organoids have been instrumental as drug screening tools in the study of SARS-CoV-2, helping to identify key pathways and potential antiviral drugs. These organoids revealed enterocytes as susceptible cells to infection. High-throughput screenings, particularly with FDA-approved drugs, have pinpointed potential inhibitors like imatinib, which is currently in clinical trials [6], as well as mycophenolic acid and quinacrine dihydrochloride. At physiological concentrations, these drugs effectively reduced infection in various organoid types [14]. A similar approach has been applied using human-airway-on-a-chip models for COVID-19 drug screening [15]. Organoids’ role in drug repurposing analysis marks a significant step in maximizing the use of existing drugs and enhancing organoid-based novel drug development. They are also pivotal in researching rare genetic diseases and testing gene therapies, with studies conducted on conditions such as Fabry disease, genetic retinal disorders, hypoplastic left heart syndrome, and microcephaly, Down syndrome, Alzheimer’s, and Parkinson’s disease [7]. Future engineering strategies should integrate biochemical and biomechanical techniques to maintain cell viability post-seeding and support cell proliferation and differentiation. The key to standardizing organoids lies in developing a unified organoid instrument to reduce variations across samples and laboratories. Furthermore, achieving a consensus in the organoid research community through robust collaboration is essential for standardization.

## 3D micropatterning augments organogenesis

**Figure 2.**
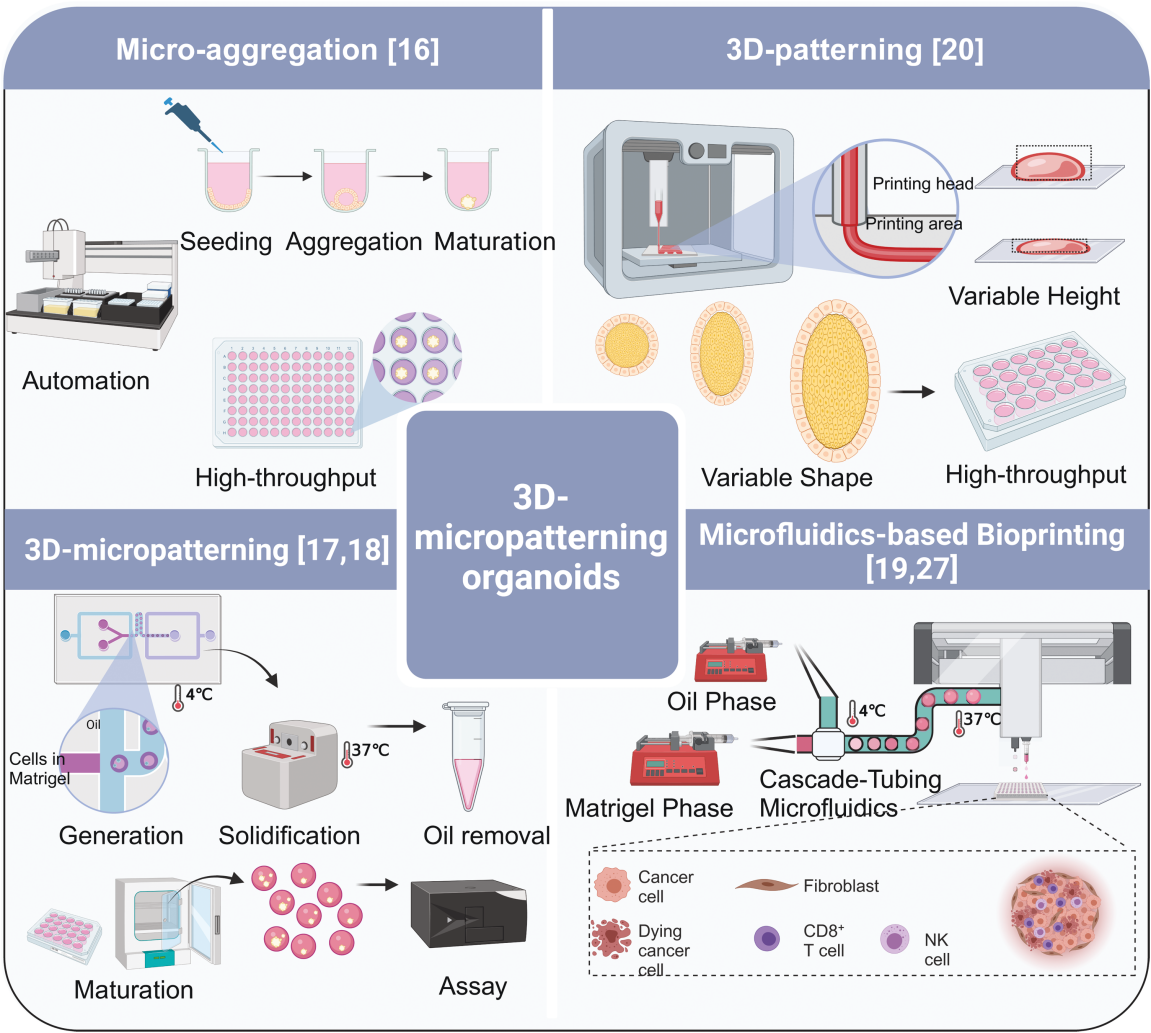
3D micropatterned organoids can both increase modeling efficiency by combining the automation platform and improve the standardization in the organoid-based clinical and pharmaceutical uses.

3D micropatterning has become a key strategy in enhancing the efficiency, quality, and standardization of organoid modeling for clinical and pharmaceutical applications. This technique focuses on the exact spatial manipulation of cells and the extracellular matrix, employing methods such as microfluidics, 3D bioprinting, etc. (Fig. 2). Effective tactics include augmenting local cell density or forming primary cell colonies. These are achieved through techniques like micro-aggregation [16, 17], droplet-encapsulation [18, 19], and bioprinting [20–22] (Table 1).

**Table 1.** Summary of different organoid fabrication techniques.

	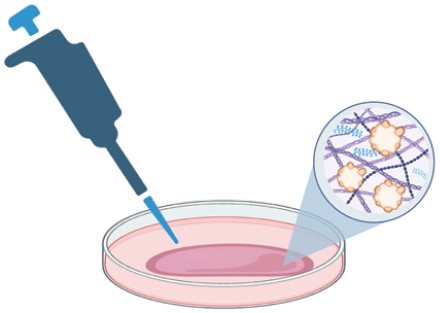	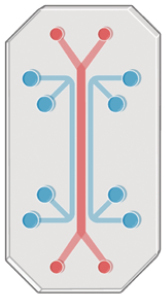	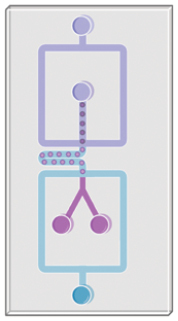	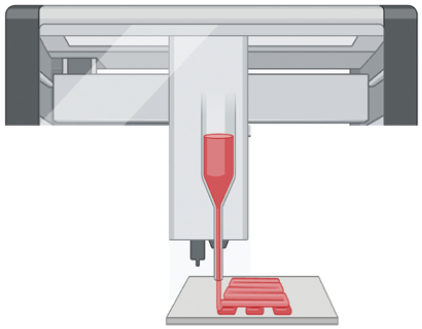	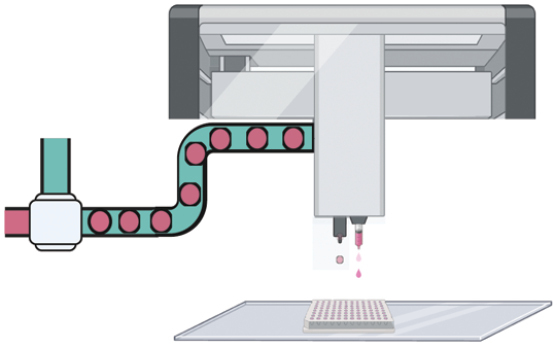	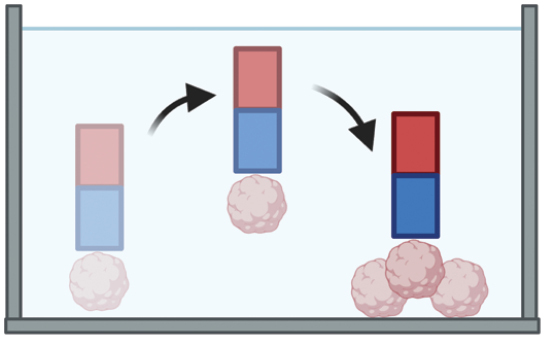
	Classical organoids	Organs-on-a-chip	Droplet-based microfluidics	Traditional 3D bioprinting	Organoid/bead-jet printing	Magnetic spatially patterned organoid transfer (SPOT)
Innovation year	2009	2010-2012	2003	2005	2020	2023
Stem cells	√	√	√	√	√	√
Primary cells	√	×	√	√	√	√
Throughput	Low	Low	High	Low	High	Low
Reproducibility	Low	Low (Primary cells)	High	High	High	Medium
Matrigel compatibility	√	√	×	×	√	×
Stromal inclusion	×	√	√	√	√	Not determined

Micro-aggregation, leveraging cells’ self-aggregation properties, employs soft lithography to create microcavities or uses liquid tension in a hanging-drop setup [23]. This process promotes cell aggregation, forming uniform spheroids or precursors. Lutolf et al. developed micro-engineered devices that confine organoids in polymer-hydrogel embedded microcavity arrays, standardizing adult stem cell cultures for industrial applications [16]. Droplet-encapsulation utilizes droplet-microfluidics, combining hydrodynamic forces and interfacial tension to form uniform, sub-microliter droplets in an immiscible fluid [24]. Our team implemented an automated drug screening method using this technique [19]. By applying micro-capillary confinement, similar to soft lithography in uniformity and throughput [18], we achieved higher cell density encapsulation without surfactants. This method encapsulated dense primary tumor cells, forming cell colonies, and producing monodisperse tumor organoids in just 7 days. However, efforts to increase production efficiency using microdroplet augmentation [18] sometimes compromise organoid growth rates and sizes due to reduced encapsulation density. Bioprinting, initially used in macro-tissue regeneration, now facilitates larger-scale organoid creation, enhancing biomimicry. This technique precisely controls geometry and cellular density, enabling the production of centimeter-scale tissues with self-organized features, such as branched vasculature, tubular intestinal epithelia, and *in vivo*-like crypts and villi domains [21].

3D micropatterning techniques show potential in achieving ‘maturation and standardization’ for laboratory-scale organoid models, yet their application in clinical and pharmaceutical settings is still evolving. Micro-extrusion, effective in kidney organoid development [25], faces limitations in production volume due to a lower threshold. Inkjet printing, while precise, struggles with maintaining structural stability in bioinks, especially in the *z*-dimension, owing to ink-drop splashes. Geometry-based patterning methods [25, 26] offer high control in organoid morphogenesis but fall short in handling primary cell derivatives and ensuring individual organoid controllability. Moreover, Matrigel, a standard scaffolding material in organoid culture, is not compatible with photo-crosslinking methods. Our team has developed “bead-jet printing” [27], significantly accelerating microbead bioprinting from around one bead per minute in aspiration-assisted methods to one bead per second. This technique differs from previous methods [28], where microbeads are manipulated in free space. Instead, we confine the microbeads within a capillary, allowing for direct printing into predefined patterns. This method facilitates rapid, ultra-soft microgel printing with precise spatial control, promising for both macro-organoid production and future organoid assembly applications.

3D micropatterning is emerging as a key technique to improve organoid modeling in terms of efficiency, quality, and standardization, crucial for clinical and pharmaceutical applications. It enhances the recapitulation of tumor microenvironmental cues, critical in regulating cancer cell responses as evidenced by clinical and experimental studies. Traditional organoid cultures, based on the growth of epithelial stem or progenitor cells without stromal interaction, often lead to inadequate phenotypic representation due to the absence of stromal elements [29–31]. However, micropatterning methods, like droplet-encapsulation and geometry-defined organoid growth [25], offer reproducible manufacturing and additional regulatory cues for differential cell organization. For example, droplet-templating creates an ECM-medium interface that guides fibroblast migration, forming a tumor-core-stroma-shell structure. Geometry-defined modeling tailors stem cell differentiation and epithelium maturation, enhancing organoid quality. Recent findings suggest moderate volumetric compression can mimic stromal effects, aiding maturation in the intestinal [32] and neuronal tissues. While 3D patterning techniques such as bespoke 3D bioprinting and lithographic micro-molding show promise in lab-scale maturation and standardization, they face challenges with production volume and structural stability. Conversely, bead-jet printing has shown the potential in speeding up microbead bioprinting, marking a promising direction for future development. Upcoming 3D patterning research should focus on complex system modeling in organoids, illuminating aspects like tumor immune barriers, blood-stream barriers, and stromal–epithelial interactions. Moreover, the vascularized 3D assembly of life-sized organs, while challenging, could revolutionize transplantation sources.

## Engineering matrix defines chemical ingredients and promotes modeling via mechano-regulation

**Figure 3.**
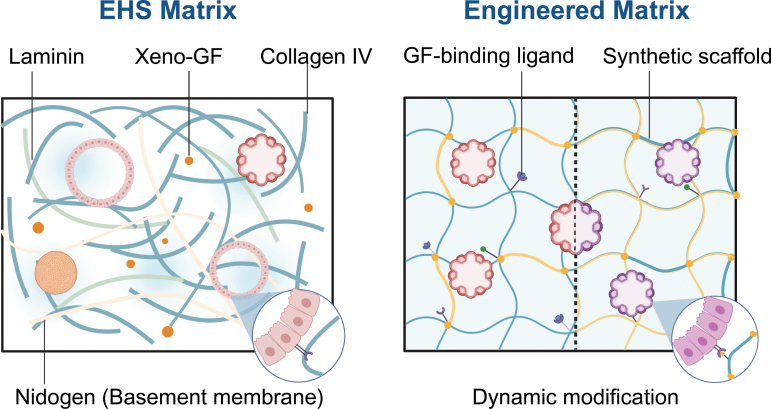
EHS-derived matrix and engineered (synthetic) matrix.

The Engelbreth–Holm–Swarm (EHS) matrix, commonly known as Matrigel or Cultrex, is the predominant standard in organoid culture due to its widespread use (Fig. 3). Its key components, laminin, collagen type IV, and nidogen, are essential for organoid formation. The EHS matrix contains over 14,000 unique peptides and nearly 2000 unique proteins, alongside growth factors, transcription factors, and cytokines [33]. This animal-derived matrix provides necessary factors for the growth of primary and stem cells. However, its complex composition poses significant challenges in studying microenvironment interactions and developmental biology. Additionally, its animal origin limits clinical applicability due to safety and scalability concerns [34]. A notable limitation is batch-to-batch variation, with only about 53% of components consistently present in each batch [33], leading to reproducibility issues. The nature of the matrix profoundly affects organoid development. Membrane-bound proteins, such as integrin, collagen, and laminin receptors, relay mechanical signals, including cell distortion and environmental stiffness, to intracellular components like TALIN or F-ACTIN. These signals are then transmitted through pathways such as FAK or Hippo to the nucleus, influencing gene expression [35–39]. Therefore, the mechanical cues from the ECM can significantly alter primary cell phenotypes.

While growth factor-reduced EHS matrices support embryonic stem cell proliferation, assessing the quality of cell-binding ligands in these matrices remains challenging. EHS matrix’s complex composition can unpredictably influence cell fate, despite its crucial role in supporting organoid cultures unsuitable for chemically defined materials. Identifying the minimal necessary matrix components is key to developing a reliable, adjustable, and clinically viable alternative. Chemical-defined hydrogels have been used for 3D *in vitro* cultures in disease modeling and implantation-based regeneration. For instance, the engineered nanofibrillar hydrogel (EKGel) successfully maintained the initiation and growth of breast cancer organoids from patient samples, with comparable histopathology, gene expression, and drug responses to parental tumors and PDOs in BME [40]. Another study introduced hyaluronan elastin-like protein (HELP) hydrogels, combining hyaluronic acid and elastin-like protein, supporting the encapsulation, proliferation, and differentiation of patient-derived intestinal organoids. This study emphasized the matrix signaling cues’ role, like RGD ligand concentration and matrix stiffness, in intestinal organoid growth and efficiency [41]. HELP hydrogels also enabled serial organoid culture for up to 12 passages with growth rates akin to EHS-matrix.

However, engineered matrices often lack the fine-tunable properties required for rapid shaping and cell-friendliness. While synthetic materials cannot fully replicate *in vivo* ECM complexity, the precise control and consistency of hydrogel systems have increased organoid culture reproducibility and modularity. Recent hydrogel systems offer adjustable mechanical, rheological, and chemical properties, focusing on non-toxicity and facilitating cell ligation, remodeling, degradation, and integration with re-synthesized substances. Lutolf et al. reported a tunable PEG-based synthetic hydrogel enhancing organoid budding and morphogenesis [42]. Sorrentino et al. developed a PEG-RGD hydrogel to model fibrotic liver mechanics, with encapsulated organoids exhibiting a fibrosis-like phenotype [43]. Despite these advances, engineered matrices with tumor-specific formulas are still not widely available commercially for organoid translation. Future research should explore further synthetic, viscoelastic gels with fast-gelling properties and cell adhesive ligands as Matrigel alternatives, potentially improving organoid consistency and clinical applicability.

## Rapid standardized drug evaluation enables personalized and high-throughput screening of drug choices

Drug response in organoids is evaluated by observing changes in morphology, cell viability, and metabolic activity pre and postdrug exposure, using multi-modality microscopic imaging with specialized devices [44]. While drugs typically target malignant tumor cells, in organoid-stroma complexes, differential cell responses to drugs are obscured by overall responses.

For organoid visualization, high-resolution microscopy, including confocal and multiphoton techniques, is essential. Organoids’ complex 3D structures necessitate advanced imaging methods, especially when using fluorescence labeling. However, fluorescent marking of specific cell types or structures in organoids can be laborious and time-intensive. High-throughput imaging is crucial for large-scale evaluation of drug efficacy and toxicity. Microfluidic systems, merged with imaging technologies, streamline organoid trapping, and analysis [16, 45]. Time-lapse live imaging further allows continuous monitoring of dynamic processes within organoids, such as cell differentiation, growth, and treatment responses.

Current imaging techniques, while advanced, encounter challenges with light scattering in small, three-dimensional tissue samples like organoids. The complex spatial organization within organoids, crucial for their development, often complicates the acquisition of clear, precise images. Traditionally, genetically engineered reporter lines were mainly available for animal-derived organoids. However, recent advancements in genome editing technologies have significantly enhanced the ability to introduce knock-ins, such as fluorescent reporters, into human organoids [46]. This development opens up new avenues for noninvasive, real-time monitoring of cell states and subcellular protein localization in human organoids. Tissue clearance methods, capable of reducing light scattering, face challenges in high-throughput screening due to their complexity. Thus, there is a demand for new, non-destructive optical techniques for deep tissue analysis. Optical coherence tomography uses infrared low scattering properties for deep tissue imaging [47]. However, as organoid data, particularly live images, accumulate, processing this information and drawing conclusions becomes more complex. To address this, deep learning is increasingly applied in organoid analysis [48]. Larsen et al. introduced a platform using high-throughput, label-free drug assays combined with neural networks and bright-field microscopy [49]. This innovative approach can predict patient-specific drug responses, particularly in solid cancers, and uniquely predicts fluorescent staining results from brightfield images. This method enhances drug evaluation by saving time and costs and reducing user errors and variations.

Beyond imaging, transcriptional profiling provides detailed insights into cell activities but is time and sample consuming. We demonstrated using a small gene library (under 200 genes) as a whole transcriptome proxy for specific predictions [50]. The process begins with screening transcriptional profiles from public databases, applying gene filters, and assessing genes for drug sensitivity influence. This library is refined using *K*-nearest neighbors cross-validation, with validation in patient-derived organoids. The digital markers, comprising tens to hundreds of gene expressions, are quantified rapidly via reverse transcription quantitative real-time PCR (qRT-PCR) within 1–3 hours post-tumor sampling. Compared to bulk RNA sequencing, our approach reduces sample and cost by at least 10-fold and time consumption by 1000-fold.

In translational research, advanced evaluation methods are being developed that combine data on morphology, viability, and metabolic activity with dynamic variations and cell-type specificity. Such comprehensive assessment requires sophisticated information acquisition equipment and the use of AI-based algorithms for feature extraction [51, 52]. Deep analysis of multi-modal organoid data and the projection of feature sensitivities are becoming increasingly crucial in this field.

## Organoids-on-a-chip

**Figure 4.**
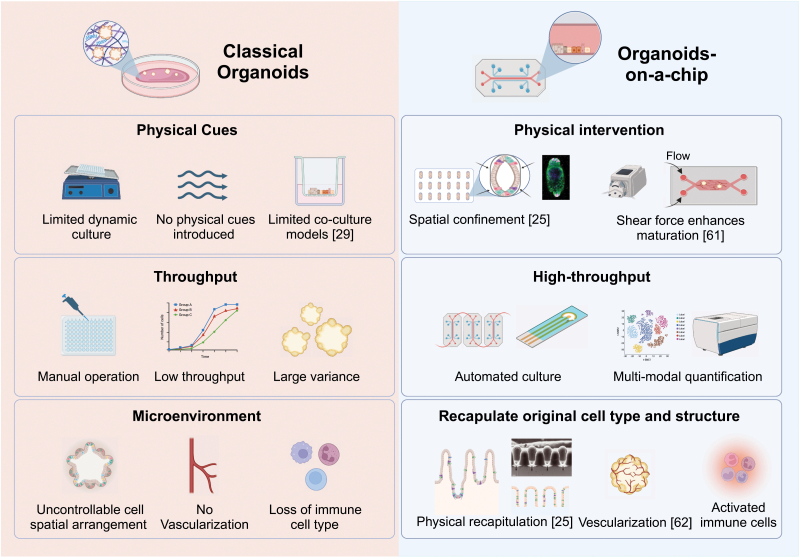
Comparison between classical organoids and organoids-on-a chip.

Organoids-on-a-chip [53] is derived from a combination of organoid and organ-on-a-chip which are fundamentally different but serve the same goal that recapitulating the microenvironment *in vivo* (Fig. 4). This hybrid model combines the intrinsic self-organization characteristic of organoids with the environmental control provided by on-chip systems. This integration enables real-time, *in situ* photonic and biochemical monitoring. The fusion of organoids and microfluidics in this manner creates a more efficient tool for a variety of applications, including drug testing, disease research, developmental biology, and other scientific and industrial pursuits.

Organoids-on-a-chip offer notable advancements over traditional plate well organoid cultures, including scalability and enhanced *in situ* observation capabilities. Crucially, they enable dynamic culturing with sheared flow or spatial pressure gradients, which is believed to enhance the growth quality of primary tissue-derived organoids. This method provides more natural conditions and improves mass transport within the tissue. This technology aligns with current research trends focusing on chronic disease treatments, particularly through the manipulation of microbiota-generated metabolites and neuronal activities. The microfluidic chip is reported to be superior for organoid culture as it actively infuses nutrients or chemicals, enabling better penetration into the organoids compared to passive diffusion [54, 55]. Specially designed chip chambers and compartments enhance organoid visualization and study mechanical effects on development [56]. Additionally, various hydrogels with specific physical properties are applied to exert compressive forces on cells or organoids [32, 57]. However, replicating the complexity of *in vivo* mechanical environments remains a challenge, limiting the study of developmental or regenerative processes *in vitro*. Progress in organoids-on-a-chip technology requires a multidisciplinary approach, combining knowledge and techniques from various fields, to advance mechanobiology studies.

Organoids-on-a-chip, integrated with a programmable flow-control system, is highly effective for automated high-throughput screening. Schuster et al. have developed an automated microfluidic system for organoid culture and analysis, enabling combinatorial and sequential drug screenings on patient-derived tumor organoids. Their research indicates that temporally modified drug regimens may be more efficacious than constant dosing or drug combinations *in vitro* [58]. However, while this system shows excellent results with adult stem cell-derived tissues, it is less adept at processing human tissue-derived primary cells. The reliance on multiple stem cell passages and purification processes may not align with the rapid modeling needs of certain clinical applications. This limitation could stem from the low adhesion properties of primary cells and the presence of impurities in tissue extracts, such as single cells, cell clusters, and extracellular matrix (ECM) debris.

Some 3D micropatterning techniques, as previously mentioned, show promise in addressing the inconsistency issues in primary organoid fabrication. Integrating organoid micropatterning with on-chip culture methods can enhance organoid reproducibility and growth quality. This combination offers a promising approach for studying organ-organ [59] and organ-microenvironment [60] interactions, as well as facilitating microscale precise interventions [61]. Tao et al. developed a multi-organoid system to replicate the human liver-pancreatic islet axis. This co-culture system precisely simulates organ–organ interactions, where glucose-stimulated insulin secretion from islet organoids enhances glucose utilization in liver organoids. Additionally, they modeled type 2 diabetes mellitus (T2DM) by subjecting this system to high glucose levels, observing mitochondrial dysfunction and impaired glucose transport in both liver and islet organoids [59].

Flow stimulation is widely used for microscale precision interventions in organoid cultures. Homan et al. [61] introduced an *in vitro* method to cultivate vascularized kidney organoids-on-a-chip using dynamic flow stimuli. This technique significantly enhances the proliferation and self-organization of endothelial progenitor cells in co-culture with kidney organoids, leading to vascular network formation. Under dynamic flow, kidney organoids show greater maturation in podocyte and tubular structures, with improved cellular polarity and gene expression typical of mature kidney tissue, exceeding the maturation levels in static conditions. Our research group has also developed co-culture organoids-on-a-chip models that host both vessels and tumoroids on a single chip. Hu et al. [62] created a perfusable, vascularized tumoroid-on-a-chip to mimic the *in vivo* tumor microenvironment. Their findings indicate that the prolyl hydroxylases (PHD) inhibitor dimethylallyl glycine not only preserves healthy blood vessels but also enhances the effectiveness of anticancer drugs like paclitaxel and cisplatin in the co-culture model.

Contrasting with traditional organs-on-a-chip, where cell interaction integrity is often low, Huh et al. developed a lung-on-a-chip microsystem that effectively recreates the critical alveolar-capillary interface of the human lung [63]. This system features epithelial and endothelial cells, separated by a thin, porous, flexible membrane coated with extracellular matrix (ECM), serving as an artificial interstitium. This on-chip model simulates responses to bacteria and inflammatory cytokines in the alveolar space, using a dual-cell-type monolayer system to closely mimic complex biological functions. The advent of organoids-on-a-chip enhances this concept by enabling intrinsic interactions among multiple cell types, thereby more accurately replicating *in vivo* biological activities. Gjorevski et al. demonstrated the application of organoids-on-a-chip for spatial and temporal control. They used localized patterning of microenvironmental mechanics and hydrogel microtopography to create organoids with precise initial sizes and shapes. This approach led to the formation of macroscopic organoids resembling the periodic crypt-villus architecture of the intestinal epithelium. Such innovations facilitate the study of complex pathophysiological processes, like intestinal cell shedding [25].

Organoids, with their compact 3D structure, can be integrated into a single multi-well plate-sized chip, significantly reducing their horizontal footprint on planar chip surfaces. This adaptation makes the chip compatible with commercial reading devices, such as plate readers or high-content imaging microscopes, thus facilitating the translational application of organoids-on-a-chip. While organoids-on-a-chip offer unique advantages compared to traditional multi-well plate cultures, there are both benefits and limitations. Dynamic culture systems replicate *in vivo* microflows, providing a realistic microenvironment for cell growth and function. However, the fluid dynamics can potentially deform organoids, impacting their growth, and functional expression. In contrast, static cultures offer stability but may fail to recreate the *in vivo*-like microenvironment, leading to uneven cell growth and differentiation. Therefore, choosing between dynamic and static culture methods should be guided by specific research objectives and cell types. Looking to the future, multi-organoids-on-a-chip with enhanced control mechanisms are poised to significantly advance the study of organ interactions and developmental biology. The integrated architecture of on-chip systems is particularly beneficial for combining various monitoring techniques, such as electrophysiological, biochemical, and photonic, in real time. Moreover, the ability of this technology to standardize organoid characterization offers the potential to generate extensive multi-modal datasets, crucial for training complex algorithms.
